# The number of hospitalisations is associated with immune parameters in patients with schizophrenia in remission.

**DOI:** 10.1192/j.eurpsy.2026.10520

**Published:** 2026-07-31

**Authors:** Z. Kozłowska, A. Turek, N. Śmierciak, M. Szwajca, W. Krzyściak, P. Donicz, K. Furman, M. Pilecki

**Affiliations:** 1 Students’ Scientific Group of Child and Adolescent Psychiatry; 2 Department of Child and Adolescent Psychiatry and Psychotherapy, Chair of Psychiatry, Faculty of Medicine; 3 Department of Medical Diagnostics, Jagiellonian University, Medical College, Krakow, Poland

## Abstract

**Introduction:**

In the population of patients with schizophrenia, the number of psychiatric hospitalisations can serve as a proxy for cumulative disease severity or recurrence of psychotic episodes, though it may also be influenced by other factors, e.g. substance abuse or psychosocial instability. Meta-analytical studies have consistently demonstrated abnormalities in immune markers, including routine blood morphology, in individuals with schizophrenia. The assessment of these markers in relation to the number of psychiatric hospitalisations may provide valuable insights into immune-related mechanisms contributing to disease burden.

**Objectives:**

The aim of this study was to investigate the relationship between clinical history parameters and levels of blood components, including blood morphology, lipid profile, hormones, and inflammatory markers, in patients with schizophrenia.

**Methods:**

Patients with diagnosed schizophrenia in remission (n=37) had their blood taken in the morning. A set of tests, including blood morphology evaluation, lipid profile, C-reactive protein (CRP), free triiodothyronine (FT3) and dehydroepiandosterone sulfate (DHEA-S), was performed in the diagnostic laboratory of the University Hospital in Krakow. Each patient underwent a detailed psychiatric interview, with additional information provided by a close relative. The results were subjected to statistical analyses, using Spearman’s rank correlation coefficient.

**Results:**

Significant positive correlations between the number of hospitalisations and white blood cells (WBC) (r = 0.42; p < 0.01), neutrophils (r = 0.34; p < 0.05), lymphocytes (r = 0.423; p < 0.01), eosinophils (r = 0.33; p < 0.05), reactivated lymphocytes (r = 0.39; p < 0.05) and CRP (r = 0.48; p < 0.01) were observed. The number of hospitalisations correlated negatively with the monocyte’s percentage related to WBC (r = −0.54; p = 0.001), FT3 (r = −0.45; p < 0.01), DHEA-S (r = −0.39; p < 0.05) and HDL (r = −0.37; p < 0.05). Importantly, no significant correlations were found between the blood parameters mentioned above and other indicators of the clinical picture, such as the duration of treatment, age of onset, frequency of hospitalisations or daily neuroleptic dosage equivalent.

**Conclusions:**

The number of psychiatric hospitalisations in schizophrenia is linked to various blood parameters, mainly immune parameters. Since the same parameters do not significantly associate with the age of onset, frequency of hospitalisations, or daily antipsychotic dosage, it is plausible that observed changes reflect trait-related immune dysregulation rather than effects of medication or illness chronicity. The findings suggest an association between the number of hospitalisations and immune parameters, although the direction of this relationship remains unclear. Further research concentrated on the distinctive aspects of psychiatric hospitalisation in schizophrenia could provide a deeper understanding of the disease.

**Disclosure of Interest:**

None Declared

